# Differences in Supraspinal and Spinal Excitability during Various Force Outputs of the Biceps Brachii in Chronic- and Non-Resistance Trained Individuals

**DOI:** 10.1371/journal.pone.0098468

**Published:** 2014-05-29

**Authors:** Gregory E. P. Pearcey, Kevin E. Power, Duane C. Button

**Affiliations:** 1 School of Human Kinetics and Recreation, Memorial University of Newfoundland, St. John's, NL, Canada; 2 Faculty of Medicine, Memorial University of Newfoundland, St. John's, NL, Canada; University of Bologna, Italy

## Abstract

Motor evoked potentials (MEP) and cervicomedullary evoked potentials (CMEP) may help determine the corticospinal adaptations underlying chronic resistance training-induced increases in voluntary force production. The purpose of the study was to determine the effect of chronic resistance training on corticospinal excitability (CE) of the biceps brachii during elbow flexion contractions at various intensities and the CNS site (i.e. supraspinal or spinal) predominantly responsible for any training-induced differences in CE. Fifteen male subjects were divided into two groups: 1) chronic resistance-trained (RT), (n = 8) and 2) non-RT, (n = 7). Each group performed four sets of ∼5 s elbow flexion contractions of the dominant arm at 10 target forces (from 10%–100% MVC). During each contraction, subjects received 1) transcranial magnetic stimulation, 2) transmastoid electrical stimulation and 3) brachial plexus electrical stimulation, to determine MEP, CMEP and compound muscle action potential (M_max_) amplitudes, respectively, of the biceps brachii. All MEP and CMEP amplitudes were normalized to M_max_. MEP amplitudes were similar in both groups up to 50% MVC, however, beyond 50% MVC, MEP amplitudes were lower in the chronic RT group (p<0.05). CMEP amplitudes recorded from 10–100% MVC were similar for both groups. The ratio of MEP amplitude/absolute force and CMEP amplitude/absolute force were reduced (p<0.012) at all contraction intensities from 10–100% MVC in the chronic-RT compared to the non-RT group. In conclusion, chronic resistance training alters supraspinal and spinal excitability. However, adaptations in the spinal cord (i.e. motoneurone) seem to have a greater influence on the altered CE.

## Introduction

Neural adaptations account for a large portion of the initial increase in strength following the commencement of a resistance training program [1]–[4]. Various stimulation techniques including transcranial magnetic stimulation (TMS), transcranial electrical stimulation (TES), transmastoid electrical stimulation (TMES), and peripheral nerve stimulation (i.e. Hoffman-reflex, H-reflex) have been used to examine these ‘neural adaptations,’ each with their own strengths and weaknesses. The results have determined that acute resistance training alters the corticospinal excitability (CE) of both upper- and lower-limb muscles, including the first dorsal interosseous (FDI) [5], [6], extensor carpi radialis brevis [7], biceps brachii [8], [9], rectus femoris [10], [11] tibialis anterior [12] and soleus [13], [14].

While it is generally accepted that initial strength gains during a resistance-training program are due to changes in CE, it is presently unclear whether the predominant site of those adaptations is of supraspinal or spinal origin, though it is likely that both are involved. TMS is often employed to assess CE. The difficulty of using TMS alone for determining changes in CE is that the amplitude of a TMS-induced motor evoked potential (MEP) could be affected anywhere along the corticospinal pathway (i.e. from corticoneurones in the brain to the motoneurones in the spinal cord). A relatively new and underutilized technique, TMES, which stimulates the corticospinal tracts independent of the corticoneurones, can be used in combination with TMS to identify whether or not changes in CE are of supraspinal or spinal origin [7], [15], [16].

As a resistance training program progresses, further increases in strength are thought to be mainly influenced by morphological adaptations of the muscle [1]. However, very few studies have examined how long-term (*chronic*) resistance training affects the manner in which the central nervous system (CNS) generates force output. Studies that have examined the tibialis anterior [17] and biceps brachii [18] suggest that there are no differences in CE between chronic resistance trained (RT) (2–3 years) and non-RT individuals. This apparent lack of change in CE may be due to the muscle group and protocols utilized. The tibialis anterior examined by Tallent and colleagues (2013) is not a muscle that individuals typically resistance train on a consistent basis and the TMS protocol used by del Olmo et al. (2006) could not differentiate between supraspinal and spinal excitability or whether the lack of change in MEP amplitude occurred due to a masking effect from increased spinal excitability. Research on a muscle that is extensively activated during resistance training (i.e. biceps brachii) combined with a stimulation protocol capable of examining both supraspinal and spinal excitability may allow further insight into the neural adaptations induced via *chronic* resistance training.

In *non-training* studies examining elbow flexion and index finger abduction, MEPs and CMEPs recorded from the biceps brachii and brachioradialis increase similarly from weak to strong contractions and then decrease at the strongest contractions (i.e. ∼>60 MVC); a response that also occurred in MEPs recorded from the FDI [19]. Oya at al. [20] also found similar MEP and CMEP responses in the soleus and medial gastrocnemius muscles. Both studies concluded that the decrease in both MEPs and CMEPs at the higher contraction intensities were due to spinal mechanisms. Only two studies have used stimulation techniques (TMS, TES and TMES) that produce MEPs and CMEPs to determine the effects of *acute* resistance training on CE and involved the FDI and extensor carpi radialis brevis [5], [7]. Both studies concluded that enhanced force production following training was probably due to changes at the spinal, not supraspinal level. It remains to be determined how CE in chronic-RT individuals is modulated over various contraction intensities, and if so, whether or not the modulation is predominantly supraspinal or spinal in origin.

The objectives of the current study were to determine: 1) the effect of *chronic* resistance training on CE of the biceps brachii during isometric elbow flexion contractions at various intensities and 2) if there was an effect, the CNS site (i.e. supraspinal or spinal) predominantly responsible for any training-induced differences in CE. Based on the work by Carroll et al. (2002, 2009), we hypothesized that CE would be altered in the biceps brachii of chronic-RT individuals during strong, but not weak elbow flexion contractions, mainly due to increased spinal excitability. A portion of the current results have been reported elsewhere in abstract form [21].

## Methods

### Subjects

Fifteen apparently healthy subjects without history of neurological disease volunteered for the study. The 15 subjects were divided into two groups consisting of 8 chronic-RT males (height 180.6±5.23 cm, weight 87.9±9.28 kg, age 24.3±2.03 years) and 7 non-RT males (height 177.7±4.34 cm, weight 72.4±10.93 kg, age 22.5±5.99 years) who were all recreationally active. All subjects were recruited from the university population. Subjects in the chronic-RT group had at least 2 continuous years (≥3 times per week) of resistance training experience. The chronic-RT group routinely performed a variety of compound, multi-jointed exercises. The subjects in the non-RT group had no resistance training experience. Subjects were verbally informed of all procedures, and if willing to participate, read and signed a written consent form. Subjects also completed a magnetic stimulation safety checklist designed [22] to screen for potential contraindications with magnetic stimulation procedures prior to the start of the experiment. The Memorial University of Newfoundland Interdisciplinary Committee on Ethics in Human Research approved the study (20131456-HK) and was accordance with the Tri-Council guideline in Canada with full disclosure of potential risks to subjects.

### Experimental Set-up

**Figure 1:**
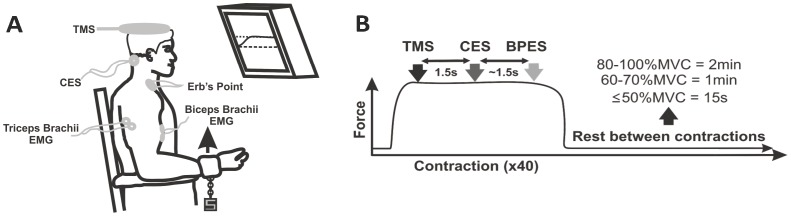
Experimental protocol. (A) Schematic diagram of experimental apparatus for elbow flexion from 10–100% MVC and stimulation location. (B) Subjects performed 4 sets of 10–100% MVCs (40 contractions in total) and received transcranial magnetic stimulation of the motor cortex (black arrow, at 1.0 s), cervicomedullary electrical stimulation of the corticospinal tracts (white arrow, at 2.5 s) and brachial plexus electrical stimulation (Erb's point) of the peripheral nerve (grey arrow, at 4.0 s) following the onset of muscle contraction. The amount of rest given between contractions depended on the contraction intensity (i.e. percentage of MVC) and is illustrated at the far right.

To determine elbow flexor forces, subjects sat in an upright position with hips, knees and elbows flexed at 90° with forearms supinated and resting on padded support. The upper torso rested against the backrest and was secured with straps around the waist and shoulders. The wrist of the dominant arm was inserted into a padded strap, attached by a high-tension wire that measured force using a load cell (Omegadyne Inc., Sunbury, OHIO). The subjects performed 5s isometric contractions with all forces detected by the load cell, which were amplified (x1000) (CED 1902) and displayed on a computer screen (Fig. 1A).

Electromyography (EMG) activity was recorded from the biceps brachii and triceps brachii muscles. Surface EMG recording electrodes (MediTrace Pellet Ag/AgCl electrodes, disc shape, and 10 mm in diameter, Graphic Controls Ltd., Buffalo, NY) were placed 2 cm apart (centre to centre) over the mid-muscle belly of the subject's biceps brachii. Since the EMG activity recorded from the three triceps brachii muscles is similar during an MVC of the elbow extensors (elbow flexed at 90 and shoulder at 0 of flexion, as performed in the current study) [23], we chose to record EMG from the lateral head of the triceps brachii muscle. A ground electrode was secured on the lateral epicondyle. Thorough skin preparation for all electrodes included shaving hair off the desired area, removal of dead epithelial cells from the desired area with abrasive sand paper, followed by cleansing with an isopropyl alcohol swab. An inter-electrode impedance of <5 kOhms was obtained prior to recording to ensure an adequate signal-to-noise ratio. EMG signals were amplified (x1000) (CED 1902) and filtered using a 3-pole Butterworth with cutoff frequencies of 10–1000 Hz. All signals were analog-digitally converted at a sampling rate of 5 KHz using a CED 1401 interface and Signal 4 software (Cambridge Electronic Design Ltd., Cambridge, UK). Recordings were made from the dominant arm for each subject.

#### Stimulation conditions

Motor responses from the biceps brachii were elicited via 1) transcranial magnetic stimulation (TMS), 2) transmastoid electrical stimulation (TMES)and 3) brachial plexus electrical stimulation at Erb's point. Stimulation intensities used for TMS and TMES were adjusted so that the evoked potentials produced by each, MEPs and CMEPs, respectively, were of similar amplitude and normalized to a maximal M-wave (M_max_). Stimulation intensities were set during an isometric elbow flexion contraction equal to 5% of MVC.

##### Transcranial magnetic stimulation

MEP responses of the biceps brachii were elicited via TMS over the motor cortex in the left or right hemisphere (depending on arm dominance) using a circular coil (13.5 cm outside diameter) attached to a Magstim 200 stimulator (Magstim, Dyfed, UK). The coil was placed horizontally over the vertex with the direction of the current flow to specifically activate the left or right cortex. To locate vertex, the distances from nasion to inion and from tragus to tragus were measured and marks were placed halfway directly on the scalp for both measurements. The intersection of both halfway marks was defined as vertex. During a 5% MVC, the stimulation intensity was altered to elicit a MEP amplitude that was between 10–20% of the M_max_ amplitude. The stimulator setting used to evoke MEP amplitude that was between 10–20% of the M_max_ amplitude was then used for the remainder of the experiment.

##### Transmastoid electrical stimulation

CMEP responses of the biceps brachii were elicited via electrical stimulation of the corticospinal tracts. Stimulation was elicited via adhesive Ag-AgCl electrodes fixed to the skin over the mastoid processes and current was passed between them (100 µs duration, 150–350 mA; model DS7AH, Digitimer Ltd, Welwyn Garden City, UK) with the anode on the right side and cathode on the left side and vice versa depending on hand dominance [24], [25]. During a 5% MVC, the stimulation intensity was altered to elicit a CMEP amplitude that matched the MEP amplitude. This stimulation intensity was used to evoke a CMEP for all contractions in the experimental protocol. TMS and TMES evoked potentials were matched in amplitude to ensure that a similar portion of the motoneurone pool was activated by each stimulus. For example, if MEPs were much larger than CMEPs, then one could suggest that the TMS response would be examining the excitability of different portions of the motoneurone pool. Close attention to the latency of the CMEPs was monitored because evoked stimulation to the mastoid processes can activate axons near the ventral roots which subsequently decreases the onset latency of the CMEP by ∼2 ms [26]. Since the onset latency of the CMEP was ∼3 ms shorter than the MEP and ∼3 ms longer than M_max_ we are confident that we were stimulating the descending corticospinal tracts.

##### Brachial plexus electrical stimulation (referred to as Erb's point hereafter)

To evoke a M_max_ in the biceps brachii, electrical stimulation was applied to Erb's point during a 5% MVC. Erb's point was electrically stimulated via adhesive Ag-AgCl electrodes (diameter 10 mm) fixed to the skin over the supraclavicular fossa (cathode) and the acromion process (anode). Current pulses (200 µs duration, 100–300 mA) were delivered via a constant current stimulator (DS7AH, Digitimer Ltd, Welwyn Garden City, UK). The electrical current was gradually increased until the M-wave of the biceps brachii no longer increased. The stimulator setting used to evoke M_max_ at 5% MVC was then used for the remainder of the experiment.

### Experimental Protocol

**Figure 2:**
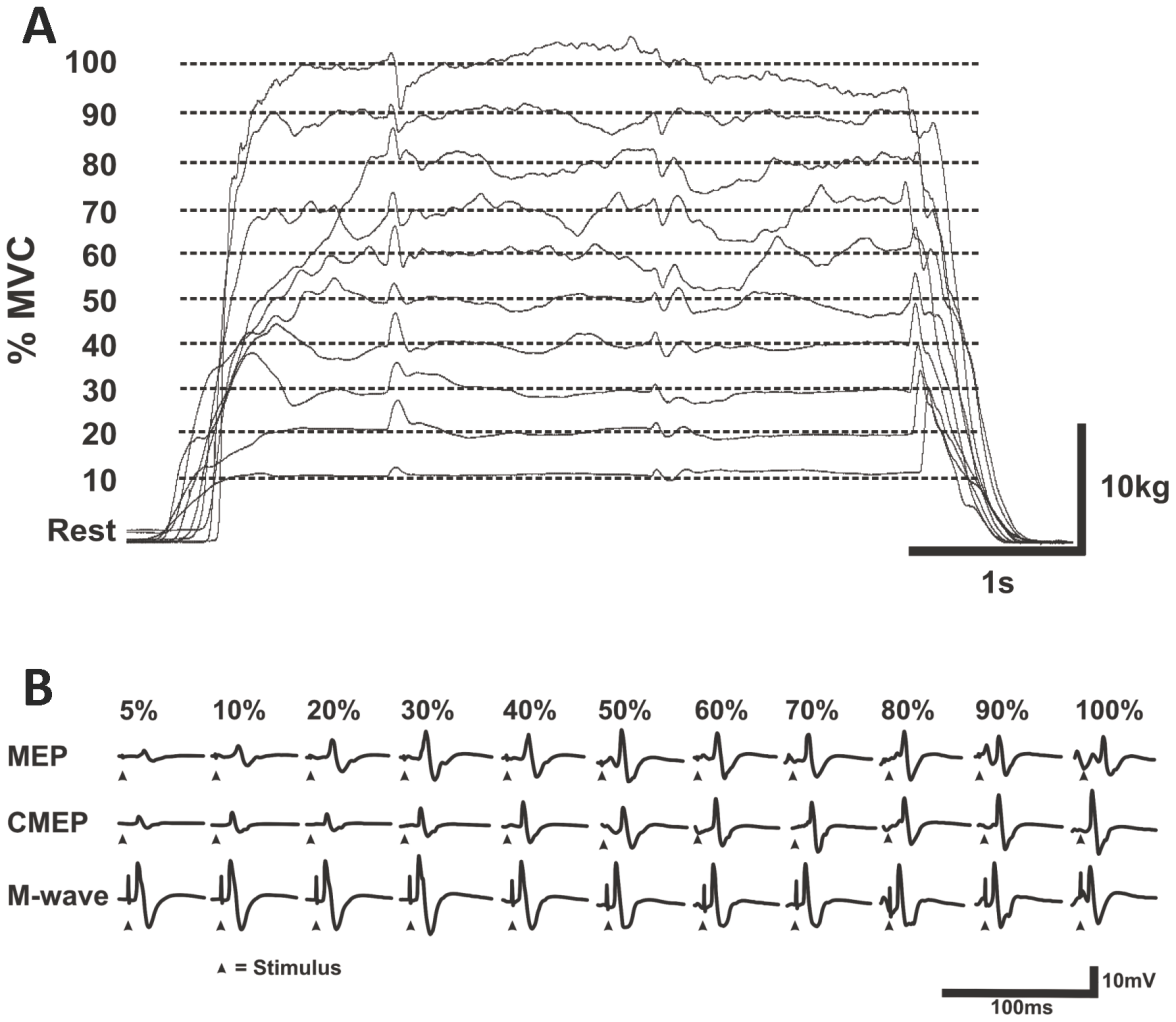
Force output from 10–100% MVC and corticospinal responses of the biceps brachii recorded from a chronic-RT subject. (A) Individual raw data traces from a single subject of one set of contractions from 10–100% MVC with the three stimuli: transcranial magnetic (MEP), cervicomedullary (CMEP) and brachial plexus stimulation (M-wave). Additional force is superimposed on the actual force following each stimulus at each contraction intensity. (B) Individual raw data traces from the same subject showing EMG responses (MEP-top, CMEP-middle, and M-wave-bottom) of the biceps brachii following each stimulus. Arrows indicate the time of stimulation.

In a single experimental session (∼1.5 hrs) subjects first performed isometric contractions for 5 s at various low intensities to get accustomed to producing varying contraction intensities. The subjects then performed an elbow flexor MVC. Following the MVC, subjects were exposed to the 3 stimulation conditions 1) Erb's point, 2) TMS and 3) TMES while performing a 5% MVC to determine the stimulation intensities to be used throughout the experiment. Once the stimulation intensities were determined, the subjects began the experimental protocol. Subjects performed a voluntary isometric contraction protocol which included four sets of 5 s contractions of the dominant elbow flexors at 10 target forces (i.e. 10, 20, 30, 40, 50, 60, 70, 80, 90, and 100% of MVC) for a total of 40 contractions (i.e. 4 contractions at each target force). For each contraction the target force and the force exerted by the subjects were displayed on a computer screen. Subjects were required to match the target force with their exerted force and maintain it for 5 s. Once the subject reached the prescribed force they received triggered TMS and TMES and a manual Erb's point stimulation at 1, 2.5, and ∼4 s, respectively (Fig. 1B). Due to the experimental set-up it was only possible to trigger 2 stimulators. At the start of each set, subjects performed a MVC and all subsequent target forces within that set were made relative to it. The order of contractions was randomized for intensities between 10 and 90% of MVC. Fig. 2 shows the raw data from one subject for all of the contraction intensities in one set (Fig. 2A) and the MEP, CMEP and M-wave responses of the bicep brachii recorded during those contractions (Fig. 2B).

Due to the high volume of contractions and potential fatigue effects, the protocol was pseudo-randomized as stated above. To further minimize the effect of fatigue there was 2 minutes of rest following 90 and 100% MVCs, 1 minute of rest following 60, 70, and 80% MVCs and at least 10 s of rest following all forces at and below 50% MVC (Fig. 1B) [20]. Immediately following the completion of the contraction protocol subjects performed one additional MVC to determine whether or not the contraction protocol induced fatigue. Verbal encouragement to match the target forces was given during all contraction intensities.

### Data Analysis and Statistics

Average biceps brachii force and average biceps and triceps brachii root mean square EMG (rmsEMG) were measured at each contraction intensity for a 1 s period between TMS and TMES stimulations. Biceps brachii rmsEMG activity recorded from 10–90% MVC was normalized to 100% MVC. Muscle co-activation was quantified by computing the ratio between the triceps/biceps rmsEMG at all contraction intensities from 10–100% MVC.

Biceps brachii MEP, CMEP and M-wave peak-to-peak amplitudes (see Table 1 for all average chronic-RT and non-RT amplitude values at each contraction intensity) and onset latencies were measured from all %MVC forces in each set. In total there were four MEP, CMEP and M-wave responses recorded and averaged for each contraction intensity. MEPs and CMEPs peak-to-peak amplitudes were normalized to M-wave amplitude at each %MVC force. Since amplitudes and areas give similar results we chose to measure the amplitudes for comparison [27], [28]. Onset latencies for MEP, CMEP and M-waves were defined as the time between the stimulus artifact and the onset of the evoked potential. Force and rmsEMG averages were also measured for 50 ms prior to each stimulus for each %MVC force. All data were analyzed off-line using Signal 4.0 software (CED, UK).

**Table 1:** Raw data for amplitudes of motor evoked potentials (MEPs), cervicomedullary motor evoked potentials (CMEPs) and muscle compound action potentials (M-wave) in chronic-RT and non-RT groups from 10–100% maximum voluntary contraction (MVC).

**Chronic-RT**										
***%MVC***	**10**	**20**	**30**	**40**	**50**	**60**	**70**	**80**	**90**	**100**
MEP Amplitude (mV)	3.3±0.75	5.0±1.02	7.3±1.31	8.0±1.57	8.1±1.71	7.6±1.57	7.4±1.45	7.0±1.41	6.8±1.44	6.4±1.49
CMEP Amplitude (mV)	2.3±0.82	2.9±0.85	4.3±1.18	5.1±1.48	5.2±1.65	5.3±1.47	5.3±1.64	5.6±1.94	6.2±1.84	6.0±1.74
M-wave Amplitude (mV)	13.0±2.51	12.3±2.33	12.3±2.50	12.1±2.32	11.5±2.20	11.5±2.12	11.7±2.21	10.9±2.26	11.5±2.47	10.6±2.39
**Non-RT**										
***Target %MVC***	**10**	**20**	**30**	**40**	**50**	**60**	**70**	**80**	**90**	**100**
MEP Amplitude (mV)	3.0±0.54	4.9±0.83	7.3±0.71	7.3±0.75	7.1±0.77	7.6±0.89	7.2±0.83	6.9±0.83	6.7±0.83	6.4±0.73
CMEP Amplitude (mV)	2.7±0.44	3.0±0.53	4.2±1.09	4.9±1.11	5.4±1.27	5.9±1.51	5.2±1.13	5.5±1.24	5.1±1.09	4.9±1.06
M-wave Amplitude (mV)	11.2±0.77	10.9±0.87	10.9±1.04	10.0±1.29	10.2±1.33	10.5±1.48	10.0±1.49	9.7±1.37	9.9±1.42	9.3±1.34

Data are reported as means±SE.

A series of one-way ANOVAs were performed to compare between group (chronic-RT vs. non-RT) differences from 10–100% MVC for all dependent variables using SPSS (SPSS 18.0 for Macintosh, IBM Corporation, Armonk, New York, USA). *F*-ratios were considered statistically significant at *p*<0.05. Descriptive statistics in text, table and figures include means ± SE.

## Results

### Control measures at 5% MVC

In the present study, stimulation intensities were determined to induce MEP and CMEP amplitudes in the biceps brachii that were ∼10–20% of M_max_ during an active contraction performed at 5% MVC. The average TMS, TMES and Erb's point stimulation intensities required to evoke MEP, CMEP and M_max_ at 5% MVC in chronic-RT and non-RT groups were 60.0±5.69% MSO and 58.8±2.35% MSO, 202.0±16.61 mA and 204.5±22.48 mA and 164.7±10.43 mA and 162.5±24.00 mA, respectively. There were no between group differences in stimulation intensities to induce, MEP (p = 0.86), CMEP (p = 0.80) and M_max_ (p = 0.95) responses in the biceps brachii.

At 5% MVC the average MEP, CMEP and M_max_, amplitudes in chronic-RT and non-RT groups were, 1.9±0.48 mV and 2.3±0.22 mV, 1.9±0.43 mV and 2.3±0.17 mV and 12.0±2.80 mV and 12.4±0.97 mV, respectively. Average MEP and CMEP amplitudes in chronic-RT and non-RT groups were ∼15.8% and 18.5% of M_max_, respectively. There were no between group differences for, MEP (p = 0.84), CMEP (p = 0.92) or M_max_ (p = 0.60) amplitudes.

There were no between group differences (p-values ranging from p = 0.31 to p = 0.71) for M_max_ amplitudes from 10–100% MVC.

The latencies from the stimulus artefact to the onset of the MEP, CMEP and M_max_ responses of the biceps brachii were measured to ensure that the supraspinal, spinal and nerve sites, respectively were being activated. MEP, CMEP and M_max_ average latencies for all subjects were 11.8±0.11 ms, 8.6±0.08 ms and 5.02±0.03 ms, respectively.

**Figure 3:**
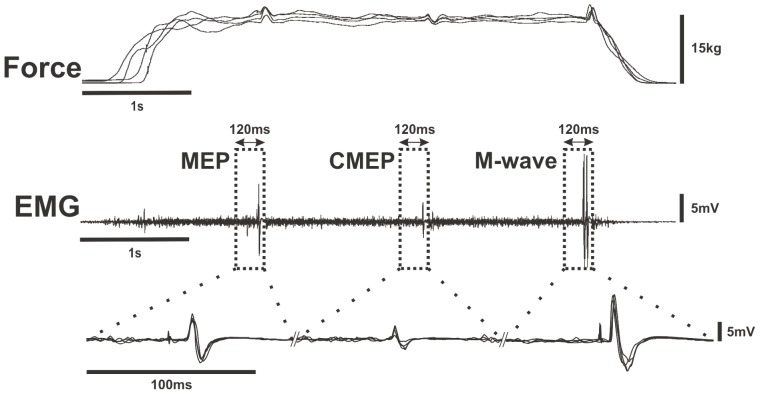
Consistency of the elbow flexion force tracing procedure and corticospinal responses in the biceps brachii within and between set contractions recorded from a chronic-RT subject. Individual raw data traces from a single subject for 4 contractions across four sets at 40% MVC (TOP). Individual raw data traces from the same subject showing EMG responses of the biceps brachii following each stimulus (transcranial magnetic - MEP, cervicomedullary - CMEP and brachial plexus - M-wave) for the 4 contractions (middle). Boxes were placed around the MEP, CMEP and M-wave and magnified for clearer illustration (bottom). MEPs, CMEPs and M-waves were very consistent within each contraction and between set contractions at the same relative intensity. Since TMS and TMES were triggered at 1 s and 2.5 s, respectively during all contraction intensities, MEPs and CMEPs overlap in the EMG Waveform. Erb's point stimulation was manually triggered at ∼4 s thus M-waves do not overlap. In the magnified M-wave waveform, M-waves from each contraction were matched by the onset of stimulus artifact.

During all contraction intensities, elbow flexion force and biceps brachii rmsEMG were measured for 50 ms prior to each stimulation type (TMS, TMES, and Erb's point) to ensure that force and background neuromuscular activity was similar within each contraction intensity and across the same relative contraction intensities (i.e. 40% MVC in sets 1–4, see Fig. 3) throughout the contraction protocol. The average pre-stimulus (pre-TMS, -TMES and -Erb's point) elbow flexion force and biceps brachii rmsEMG values were similar (p≥0.14) within and across all contraction intensities (10–100% MVC) throughout the contraction protocol.

### Elbow flexor force output in chronic-RT and non-RT

**Figure 4:**
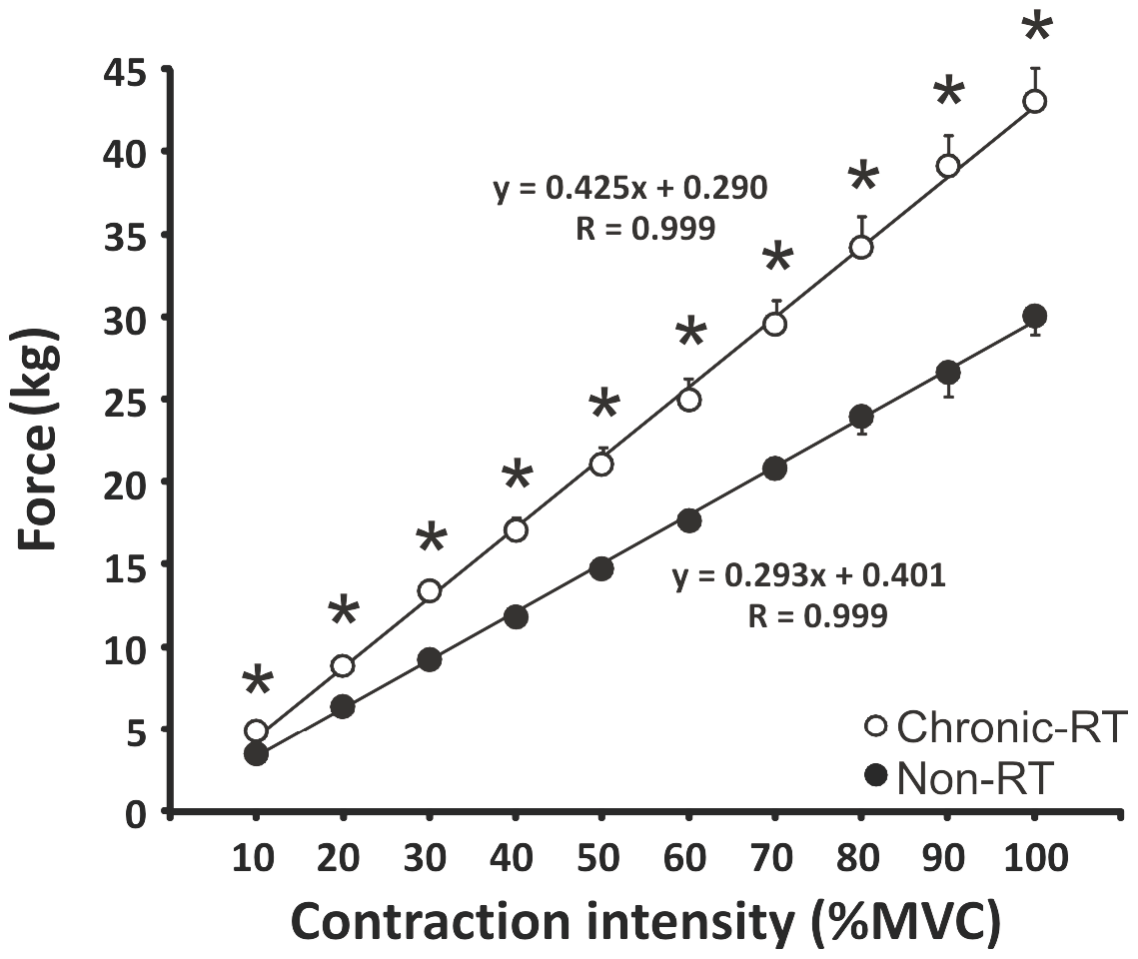
Elbow flexion force over all contraction intensities. Absolute-relative target force relationship of the elbow flexors. The slopes and r values are illustrated for each group. Each data point represents the group means ± SE. * Indicates significant (p≤0.032) differences between groups.

Overall, the chronic-RT group was able to exert 30% more force at 100% MVC than the non-RT group (p<0.001). Also, the absolute elbow flexor forces at all percentages of MVC were greater (p<0.001) in the chronic-RT group. The slopes of the absolute-relative target force relationship were 0.425 (r = 0.99) and 0.293 (r = 0.99) in the chronic-RT and non-RT groups, respectively (Fig. 4). As the relative target force increased the difference in absolute forces between the chronic-RT and non-RT groups became greater.

### Corticospinal excitability of the biceps brachii during relative force outputs

**Figure 5:**
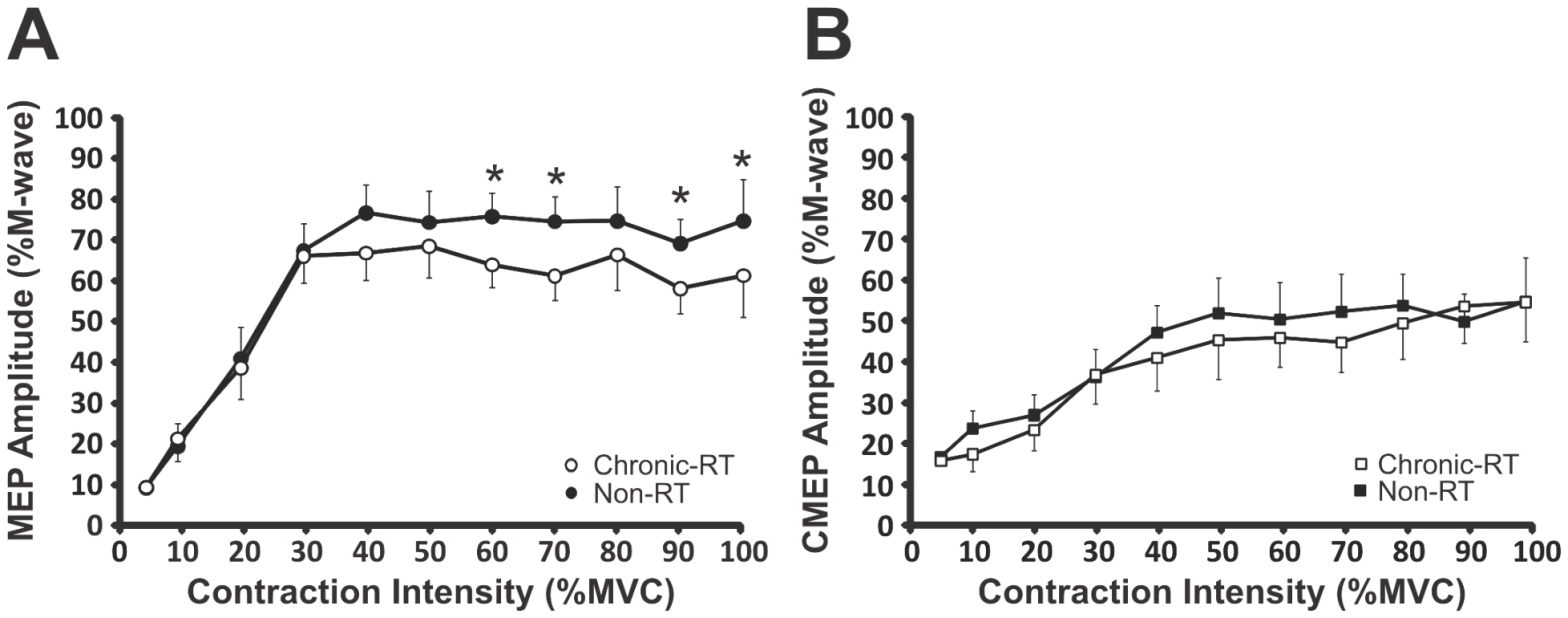
Between group differences in corticospinal responses (A) MEPs and (B) CMEPs of the biceps brachii during elbow flexion from 10-100% MVC. Each data point represents group means ± SE. * Indicates a significant (p≤0.043) difference between groups.

MEP amplitudes recorded from the chronic-RT group were 14.5% (p = 0.043), 15% (p = 0.022), 14.3% (p = 0.041), 16% (p = 0.023) less than the non-RT group at 60, 70, 90 and 100% MVC, respectively (Fig. 5A). CMEP amplitudes recorded from chronic-RT and non-RT groups were not different (p≤0.22) (Fig. 5B). There were no between group differences for MEP (chronic-RT, 11.9±0.10 ms and non-RT, 11.6±0.18 ms, p = 0.15) and CMEP (chronic-RT, 8.7±0.09 ms and non-RT, 8.6±0.15 ms, p = 0.69) onset latencies at any contraction intensity.

### Corticospinal excitability of the biceps brachii during absolute force outputs

To determine the change in MEP and CMEP responses between chronic-RT and non-RT over each absolute force output we made a ratio of MEP and CMEP to the average absolute background force 50 ms prior to each stimulus at every target force, as per Carroll et al. [5].

**Figure 6:**
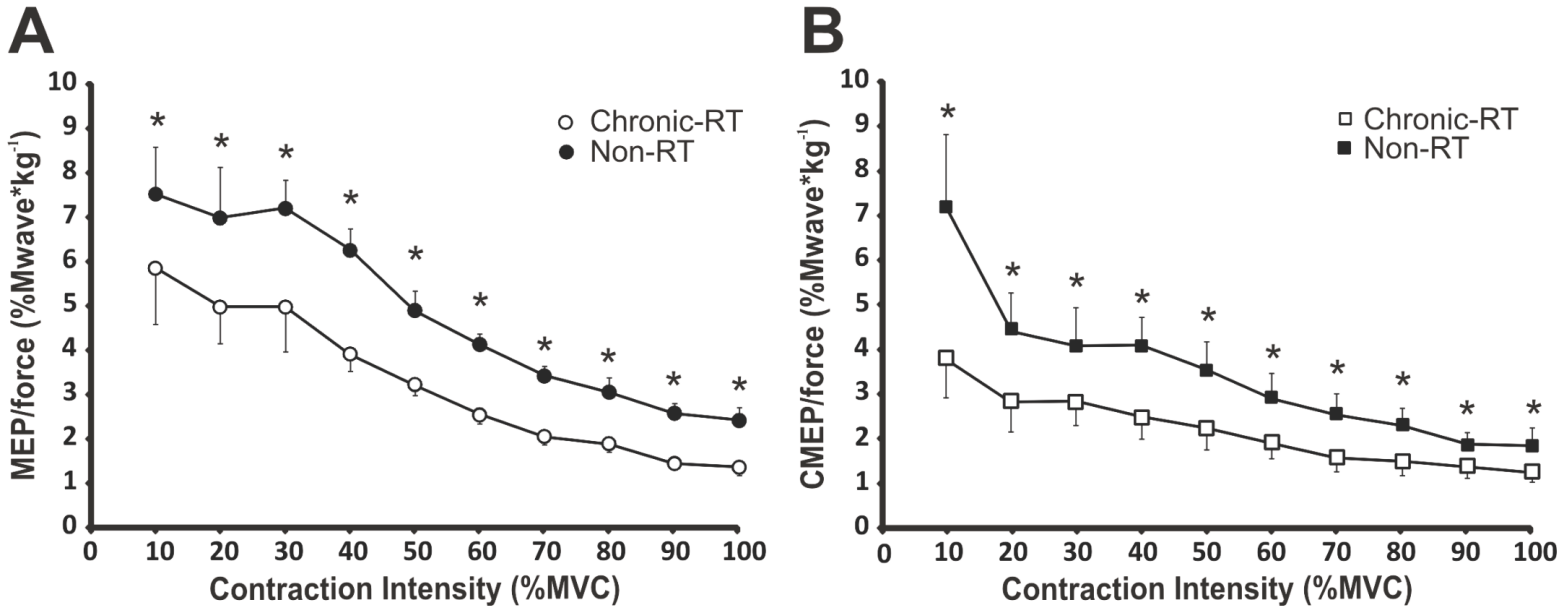
Between group differences in corticospinal responses (A) MEPs and (B) CMEPs of the biceps brachii normalized to absolute elbow flexion forces from 10–100% MVC. Each data point represents group means ± SE. * Indicates a significant (p≤0.015) difference between groups.

The ratio of MEP amplitude to absolute force recorded from the chronic-RT group was reduced, by 26 to 42% (p-values ranging from p<0.007 to p<0.001) from 10–100% MVC compared to the non-RT group (Fig. 6A). The ratio of CMEP amplitude to absolute force recorded from the chronic-RT group was also reduced by 25 to 46% (p-values ranging from p = 0.011 to p<0.001) from 10–100% MVC compared to the non-RT group (Fig. 6B).

### Biceps brachii activation and triceps:biceps coactivation ratios in chronic-RT and non-RT

**Figure 7:**
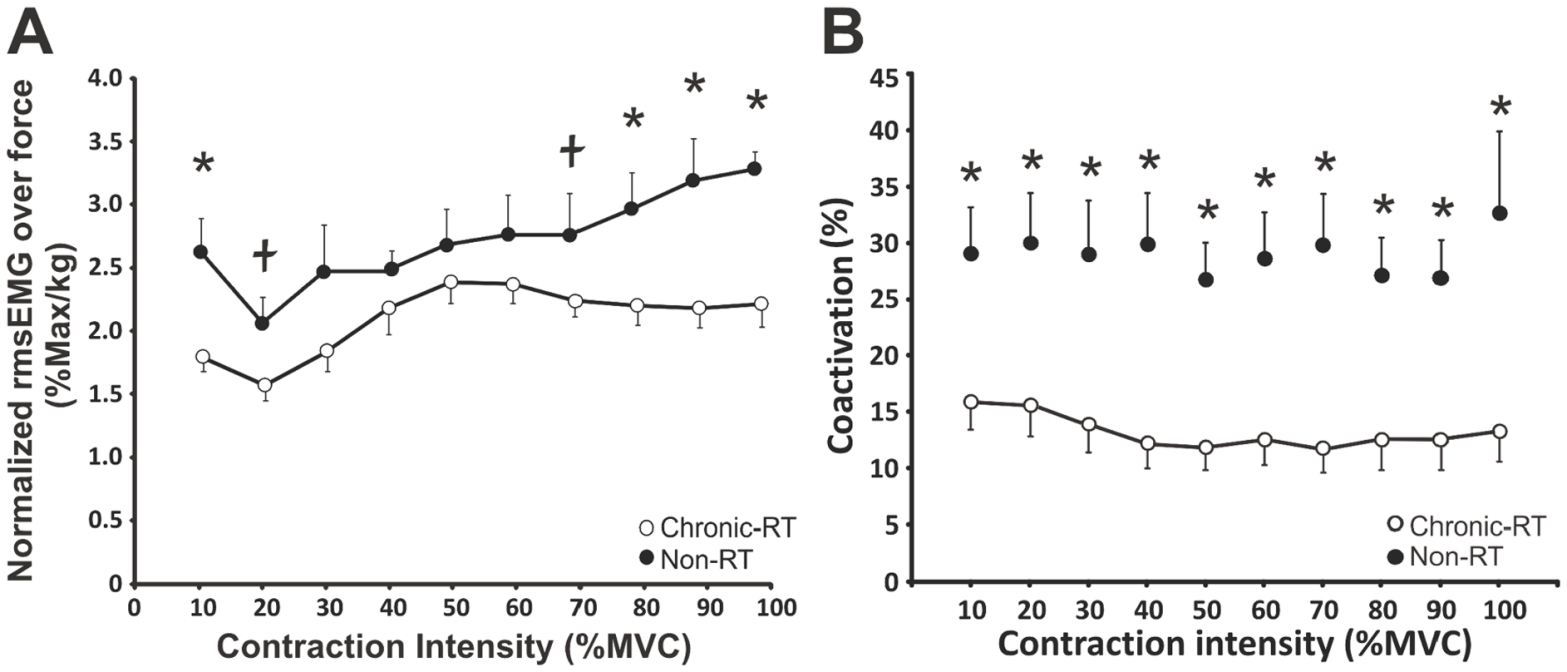
Biceps brachii activation during muscle contraction from 10–100% MVC. (A) Normalized rmsEMG to absolute elbow flexion forces recorded from 10–100%MVC. (B) Muscle coactivation (EMG of triceps:biceps brachii) 10–100% MVC. Each data point represents group means ± SE. * Indicates a significant (p≤0.041 and p<0.001 for activation and coactivation, respectively) difference between groups. Ł Indicates a trend (p≤0.075) for between group differences.

To determine the change in normalized biceps brachii rmsEMG between chronic-RT and non-RT over each absolute force output we made a ratio of normalized rmsEMG to the average absolute force recorded during each target force. The normalized rmsEMG to the absolute force ratio was higher (ranging from 12 to 45%) in the non-RT than chronic-RT from 10–100% MVC, but only statistically higher by 29.7% (p = 0.016), 31.9% (p = 0.041), 42.9% (p = 0.019 and 44.9% (p = 0.001) at 10, 80, 90, and 100% MVC, respectively. There was also a trend (p = 0.077 and 0.071) for it to be higher at 20 and 70% MVC, respectively (Fig. 7A).

The chronic-RT group had lower tricep:bicep coactivation ratios by 45–60% (p-values ranging from p = 0.003 to 0.032) over all contraction intensities compared to the non-RT group (Fig. 7B).

## Discussion

This is the first study to clearly demonstrate that the increased force generating capabilities of *chronically* resistance-trained individuals is accompanied by corticospinal plasticity. MEP amplitudes were smaller in the chronic-RT group during elbow flexion forces >50% of MVC when compared to the non-RT group, whereas CMEP amplitudes did not differ between groups. This implies that at higher contraction intensities supraspinal excitability is lower in chronic-RT individuals while spinal excitability is similar when compared to non-RT individuals. However, even with the techniques utilized it is difficult to determine whether the observed difference in MEP amplitude following *chronic* resistance training is due to predominantly supraspinal or spinal mechanisms.

In the present study it is possible that: 1) supraspinal excitability was similar between groups and spinal excitability was higher in chronic-RT individuals. MEP amplitudes were decreased in chronic-RT individuals because increased firing frequencies of the spinal motoneurones masked, to a small extent, the actual size of the MEP; 2) supraspinal and spinal excitability were increased in chronic-RT individuals. MEP amplitudes were decreased in chronic-RT individuals because increased firing frequencies of the spinal motoneurones masked, to a great extent, the actual size of the MEP; 3) supraspinal excitability was decreased in chronic-RT individuals but compensated for by an increased firing frequency of the spinal motoneurones which may allow for a reduction in supraspinal drive required for force production and movement. We suggest that the commonality between all three scenarios is an increase in the firing rate of the spinal motoneurone following *chronic* resistance training.

It is currently unknown whether the neural adaptations that accompany the commencement of a resistance training program are the same, maintained and (or) amplified in chronic-RT individuals. There are two research studies on *acute* resistance training, however, that support the current findings. Carroll et al. [5] found that a 4-week resistance training program of the index finger abductor increases strength while significantly reducing FDI MEP amplitudes (via TMS and TES) when assessed between 40–60% of MVC. Since all motor units in the FDI muscle are recruited by ∼50% of MVC [29], [30] they did not assess MEPs and CMEPs beyond 60% MVC. Carroll et al. (2009) subsequently showed a reduction in MEP and CMEP amplitudes at 50 and 75% of MVC in the extensor carpi radialis brevis following 4 weeks of resistance training. They suggest that the decreased MEP amplitude following training is most likely due to training-induced spinal motoneurone adaptations such as an increased firing rate and (or) modulation of motoneurone intrinsic properties. Changes in the after-hyperpolarization potential (AHP) (i.e. increased duration and (or) amplitude) [31] and (or) higher firing rates of the spinal motoneurone [32], [33] may reduce the probability of a motoneurone to respond to TMS, subsequently “masking” the MEP response.

Though it is not known if or how the AHP changes in response to resistance training, increased motoneurone AHP amplitude has been shown in animals following endurance training [34] and motor training [35]. In humans, increased strength following dynamic resistance training has been shown to be due to production of extra doublets and increased maximal firing rate [36], [37] leading to temporal summation of force output. Other biophysical properties of the spinal motoneurone can also be modified. Following an endurance training program, spinal motoneurones are characterized by a lowering of the voltage threshold for action potential initiation and a decreased spike rise time [38], both of which could increase firing frequency. Activation of persistent inward currents can also significantly increase and maintain motoneurone firing frequency in the presence of lower synaptic input in animals and humans [39]–[42]. Although it is not known how training affects persistent inward currents, it is thought that these currents will be strongly modulated via the monoaminergic system during exercise [43], [44], subsequently increasing motoneurone firing frequency and enhancing force. If motoneurone properties were altered following resistance training in a similar manner to the aforementioned putative mechanisms, the result would be a reduction in the effort required for their activation and enhanced firing frequencies. This could lead to increased force output. On the other hand, recent research has shown activity-dependent depressions of F-waves (following various duration maximal effort contractions in the tibialis anterior and abductor digiti minimi illustrating decreases in the excitability of the initial segment and (or) soma-dendritic membrane [45], [46] which would lead to a decreased force output. However, the effect of *chronic* resistance training on these depressions and the excitability of the initial segment or soma-dendritic membrane is unknown.

Based on our findings and those of others, there is a strong argument for an increased motoneurone firing frequency at high force output (i.e. >50% MVC) in the chronic-RT group. It is inconclusive if *chronic* resistance training affected spinal motoneurone recruitment patterns during force production because CMEPs did not differ between groups. CMEP amplitudes were recorded from the biceps brachii from 10–100% MVC as a measure of motoneurone recruitment because motor units of the biceps brachii are recruited up to and beyond 90% MVC [29]. The effects of resistance training on motor unit recruitment are relatively unknown. In animals, endurance training decreased the current required to discharge a motoneurone to fire but not the amount of current required to elicit an action potential 50% of the time (i.e. rheobase current) [47]. In humans, Van Cutsem et al. [36] showed a shift in tibialis anterior motor unit recruitment thresholds (i.e. earlier activation) via needle electrodes following resistance training. Adam et al., (1998) demonstrated that the FDI of the dominant hand had lower motor unit recruitment thresholds compared to the FDI of the non-dominant hand. Thus, the voltage threshold or recruitment threshold of spinal motoneurones can be altered. If similar mechanisms were at play in the present study one would have expected the CMEP amplitudes to have been higher in the chronic-RT group at lower contraction intensities when compared to the non-RT group, perhaps due the clustering of motoneurone thresholds. This did not occur but might be a result of the TMES protocol used, which may not have been sensitive enough to detect this type of adaptation. The TMES current used was not set to find CMEP threshold, rather it was set to induce a CMEP amplitude that was ∼15–20% of M_max_. A higher TMES current could have recruited motoneurones that would have not been recruited at a threshold current giving similar CMEP amplitudes in both groups over all relative contraction intensities. Nonetheless, altered motoneurone properties following resistance training can account for some of the increased strength that occurs with resistance training.

Following resistance training, supraspinal excitability has been shown to increase, not change, or decrease, with concomitant increases in strength. Increased supraspinal excitability following an *acute* resistance training program has been demonstrated in the tibialis anterior [12], soleus [14], rectus femoris [10], [11] and biceps brachii [8] muscles, possibly due to reduced intracortical inhibition [10], [11]. Other studies have shown no difference in supraspinal excitability following *chronic* resistance training of the tibialis anterior [17] or *acute* resistance training of the FDI [6] and biceps brachii [18] muscles. In fact, Jensen et al. (2005) actually showed decreased supraspinal excitability of the biceps brachii muscle following *acute* resistance training. A decrease in supraspinal excitability could potentially occur since resistance training attenuates movement-related cortical potentials [48] thereby enhancing the neural economy within the connections between the neurons in the cortex. These contradictory results may be a result of differences in the 1) muscles examined, 2) contraction protocols, 3) type and combination of stimulation techniques used (see review by McNeil et al. [49] for details), 4) stimulation protocol and 5) complexity of the resistance training movements [3]. Unfortunately, none of these aforementioned studies used TMES (i.e. CMEPs) to determine the impact that training induced changes in spinal motoneurones would have on CE. It has been suggested that TMS and TMES combined have advantages over other stimulation techniques in determining the CNS sites for changes in CE (see reviews by Martin et al. [16], McNeil et al. [49] and Carroll et al. [3] for more detail). There are now three studies (the present study, and Carroll et al, 2002 and 2009) that employed both TMS and TMES to determine whether CE is altered predominantly at the supraspinal or spinal level following resistance training. All three studies showed similar results (increased strength and similar MEP and CMEP responses) using different stimulation protocols on different muscles following very different training programs. Essentially the three studies illustrate that at higher relative contraction intensities (i.e. >50% MVC) altered CE appears to be predominately influenced by spinal mechanisms.

Irrespective of training status there appears to be a shift from supraspinal to spinal control of force output at relative contraction intensities >50% of MVC. In both groups, the MEP (Fig. 5A) amplitude increases to a greater degree than the CMEP (Fig. 5B) amplitude from 5–40% MVC and then plateaus. The greater increase in MEP amplitude compared to CMEP amplitude from 10–40% MVC would indicate that CE was predominantly supraspinally mediated. Following 50% MVC both MEP and CMEP amplitudes plateaued indicating that at higher contraction intensities any change in CE was predominantly spinally mediated. In non-training studies, increased CE at the supraspinal level during weak contractions (<50%MVC) has also been reported [50]–[53]. Martin et al. [19] also demonstrated that MEPs and CMEPs of the biceps brachii during strong contractions (>50% MVC) changed similarly, suggesting that spinal mechanisms (i.e. the motoneurone) are responsible for changes in the evoked potentials. Interestingly, the biceps MEP amplitude-force curve (Figure 5A) shown here (increase in MEP amplitude up to 30–40% MVC) is not similar to that found previously (increase in MEP amplitude up to 80% MVC [54] rather it was similar to that found in a rate coding muscle such as the abductor digiti minimi [54]. It is possible that the stimulation intensity used by Gelli et al. (2007), was lower, thus allowing a more sensitive measure for MEPs (smaller increments in MEP amplitude without a ‘ceiling effect’). However, this is speculative and cannot be concluded with certainty.

MEPs and CMEPs were decreased in the chronic-RT compared to the non-RT group at all absolute forces from 10–100% MVC indicating that both supraspinal and spinal excitability were lower with chronic resistance training per given amount of force generated. Carroll et al, (2002) and (2009) showed the same results after only four weeks of resistance training. In the chronic-RT group the increase in absolute force at all contraction intensities was due to neural and (or) muscle adaptations (see review by Folland and Williams [1], for details). Although we did not assess muscle girth or fat-free body mass, the chronic-RT group was ∼15 kg heavier than the non-RT group, likely due, at least in part, to increased muscle mass. Thus, following chronic resistance training, the enhanced force production was probably due to a combination of nervous system and muscular adaptations.

The chronic-RT group also had a lower absolute force-agonist EMG relationship. Biceps brachii EMG activity normalized to force was lower at every (although not statistically, see Fig. 7A) contraction intensity from 10–100% MVC in the chronic-RT group. Other studies have found a shift in the force–agonist EMG relationship (i.e., greater force for the same level of activation) following chronic resistance training (2–6 months) [55]–[57]. In the present study, the chronic-RT group also had decreased triceps brachii:biceps brachii coactivation ratios from 10–100% MVC compared to the non-RT group. On average, triceps activation was ∼15% of biceps activation in the chronic-RT group whereas it was ∼30% in the non-RT group (Fig. 7B) and these percentages were similar across all contraction intensities. Decreased co-activation has been shown to occur as a result of resistance training [37], [58]–[60] which may be due to increased sensitivity to descending motor commands at the spinal level or a decrease in reciprocal inhibition [61]. A shift in the force-agonist EMG relationship and decreased muscle coactivation would make the agonist muscle more efficient during a given contraction intensity and potentially reduce the amount of CE required to produce force.

The current study has several potential limitations. Based on our findings and others we suggest that there is an enhanced motoneurone firing frequency as a result of chronic resistance training. However, the stimulation techniques employed here cannot decipher whether this enhanced firing frequency is due to intrinsic properties of the motoneurone or changes to pre-motoneuronal sites (i.e. cortico-motoneuronal synapse). Indeed, the H-reflex is potentiated by resistance training, illustrating a pre-motoneuronal and/or motoneuronal adaptation [13]. The methodological protocol employed in the current study may have influenced the results, though we don't think this is the case. The order of stimulation techniques (i.e. MEP, CMEP, M-wave) was not randomized from contraction to contraction. Since the force and EMG prior to each stimulus were not different within each contraction the stimuli should not have affected the amplitude of the MEP or CMEP. Furthermore, M-wave did not differ between groups at any contraction intensity, thus MEP and CMEP amplitudes were likely due to changes at the supraspinal or spinal level and not due to differences in excitation across the muscle fibres. Fatigue related to the number of contractions performed throughout the protocol probably did not affect MEP and CMEP amplitudes either since the MEP and CMEP amplitudes did not differ from set to set at the same contraction intensity (see Fig. 3) and perhaps most telling, there was no significant difference between the pre- and post-contraction protocol MVCs (p = 0.28). All MEP and CMEP amplitudes recorded at a very low contraction intensity (5% MVC) were matched and did not differ between groups. Finally, differences in CE between groups were probably not due to changes in conduction speed or synaptic transmission along the corticospinal pathway because there were no differences between MEP and CMEP onset-latencies between groups at any contraction intensity.

In conclusion, enhanced strength resulting from chronic resistance training is in part due to altered CE. The predominant site for the altered CE is probably at the motoneurone. This was evidenced by a decreased MEP amplitude in chronic-RT compared to non-RT individuals at relative forces >50% MVC. Both MEP and CMEP amplitudes normalized to absolute force were lower in the chronic-RT group at all contraction intensities from 10–100% MVC. Thus, any training-induced enhancement in force production will allow for reduced corticospinal drive. A shift in the force-agoinst EMG relationship and decreased coactivation also occurred in the chronic-RT group and may contribute to the reduction in CE. *Chronic* resistance training substantially increases force output in part due to altered CE supraspinally and especially spinally (i.e. the spinal motoneurone).
